# In this month’s Bulletin

**DOI:** 10.2471/BLT.26.000726

**Published:** 2026-07-01

In the editorial section, Herry Susanto et al. (442) outline preparedness requirements for the current Bundibugyo virus disease outbreak.

## Ethiopia

### What works in financing cataract care 

Dominque Geoffrion et al. (505–510) evaluate investments in surgical provision.

## Nepal 

### Solar power for maternal and neonatal care

Samriddha Rana et al. (511–516) track lasting improvements.

## Spain

### Appropriate waste management for asthma inhalers 

Noé Garin et al. (454–464) study behaviour change on disposal methods.

## United Kingdom of Great Britain and Northern Ireland

### Clinical evaluation of infectious disease diagnostics

Edward Blandford et al. (495–504) present an umbrella protocol.

## Viet Nam

### Facility-based tuberculosis screening 

Luan Nguyen Quang Vo et al. (465–474) describe a public–private partnership.

## Global

### Regional coordinators for WHO Member States

Nikica Daraboš et al. (445–453) explore diplomatic roles.

### Tobacco control and alcohol consumption

Prashant Kumar Singh et al. (475–485) list ways to apply proven measures.

### Global health 2050

Joachim Sturmberg et al. (486–494) lay out elements of a systemic approach.

### Improved access to biosimilars

Hye-Na Kang et al. (517–521) propose regulatory changes.

### Digital health investments

Kirsten M Mathieson and Mathilde Forslund (522–524) argue for standard tracking measures.

